# Osteoporosis Management for Shoulder Surgeons

**DOI:** 10.1007/s12178-024-09927-6

**Published:** 2024-09-14

**Authors:** Kelsey M. Healy, Jacob Ritter, Emily Barr, Jessica L. Churchill, Nicholas A. Trasolini, Brian R. Waterman, Alan W. Reynolds

**Affiliations:** 1 Department of Orthopaedic Surgery and Rehabilitation, Wake Forest University School of Medicine, Winston-Salem, USA; 2 Campbell University School of Osteopathic Medicine, Lillington, USA; 3 Department of Orthopaedic Surgery, Denver Health, Denver, USA

**Keywords:** Shoulder, Surgery, Arthroplasty, Rotator Cuff, Osteoporosis, Fracture

## Abstract

**Purpose of Review:**

The aim of this review is to aggregate currently available literature as it pertains to treating surgical shoulder pathology in patients with osteoporosis.

**Recent Findings:**

Emerging data surrounding perioperative use of anti-osteoporosis medications for patients undergoing shoulder surgery have not shown definitively favorable or unfavorable outcomes. Similar evaluations in animal studies have shown promising results as a biologic augment to tendon and bone healing, especially with newer, anabolic agents.

**Summary:**

The mainstay of bone health management remains pre-operative evaluation, using opportunistic radiographic and CT based validated measurements, along with optimization of risk factors. Surgical techniques continue to incorporate implants that perform well in osteopenic bone. Promising pre-clinical studies have identified anabolic anti-osteoporosis medications as viable biologic augments to shoulder surgery, which has not been borne out in any clinical studies at this time.

## Introduction

Osteoporosis has presented itself as a growing problem for aging populations, both in the United States and worldwide, with over 10 million Americans and over 200 million women worldwide diagnosed with this bone disorder [1, 2]. As the number of aging adults continues to grow, the prevalence of osteoporosis and its health care, economic and social burdens continue to increase significantly [3]. Appropriate awareness and treatment of osteoporosis is crucial not only to prevent fractures and associated morbidity, but also to optimize surgical outcomes and overall quality of life for our patients.

Osteoporosis is common among patients who present to orthopaedic surgeons with an indication for shoulder surgery. Approximately 80% of operative proximal humerus fractures occur in women aged 74 to 84 years old [4]. Among patients proceeding with shoulder arthroplasty, at least 14.3% of those undergoing anatomic total shoulder arthroplasty and 26.2% of those undergoing reverse shoulder arthroplasty have preoperative osteoporosis [5]. With over 250,000 surgical repairs performed in the United States each year, rotator cuff tears are another common shoulder condition in this aging demographic, and quality of bone in the greater tuberosity known to play a crucial role in the durability of these repairs [6–9].

Osteoporosis is a leading risk factor for development of shoulder pathology requiring surgical intervention, and is implicated in surgical complications for all common shoulder procedures. Osteoporosis is the cause of fragility fractures; patients with osteoporotic bone are 2.6-fold more likely to incur proximal humerus fractures than those with non-osteoporotic bone [4, 10] The compromised bone quality, characterized by fragmentation, thinning of the cortical layer, and cancellous bone, also complicates osteosynthesis when surgical management is undertaken [4]. In terms of shoulder arthroplasty, osteoporosis increases the odds of peri-prosthetic fractures by 2.24 times, and prosthetic joint infections by 1.68 times, compared to non-osteoporotic controls [11–13]. Additionally, osteoporosis is an independent risk factor for acromial stress and scapular spine fractures after reverse shoulder arthroplasty [11, 14, 15]. Rotator cuff repairs are also highly dependent on overall, and especially tuberosity, bone quality for repair integrity and anchor pullout strength, with numerous studies identifying poor bone quality as a risk factor for repair failure and worse outcomes [7–9, 16].

Optimizing surgical outcomes and patient care of these shoulder conditions should routinely include consideration of bone quality, and in some cases, formal evaluation and treatment of it. The goals of this review are to synthesize the currently available data and offer evidence-based recommendations surrounding bone mineral density, as they apply to the shoulder surgeon and their patients.

### Preoperative Evaluation and Optimization

A multidisciplinary approach to management of osteoporosis is essential due to the extensive number of risk factors. Modifiable risk factors include, but are not limited to the following: inadequate nutrition, lack of physical activity, smoking, weight loss, and alcohol use [17, 18]. Current management for osteoporosis involves mitigating these risk factors through lifestyle modifications in addition to vitamin D and calcium supplementation [19]. All patients undergoing shoulder surgery should be counseled on lifestyle factors as modifiable risk factors. Extending to pharmacologic treatment pre-operatively depends on many factors. Unfortunately, many shoulder surgery candidates who would benefit from osteoporosis screening or treatment do not receive it. Despite up to two thirds of patients undergoing shoulder arthroplasty having an osteoporotic bone mineral density (BMD), only 12% have been found to have had a pre-operative BMD evaluation [20]. Screening and treatment for osteoporosis has also been found to be severely underutilized in patients undergoing rotator cuff repair [21].

The gold standard diagnostic tool for osteoporosis includes a dual-energy X-ray absorptiometry (DXA) which measures BMD at various anatomical locations, most commonly the femoral neck, and compares an individual’s density to standardized results from healthy individuals aged 25 to 35 years old. A t-score between -1 to -2.5 standard deviations (SD) below the mean indicates a BMD consistent with osteopenia and a t-score beyond -2.5 SD below the mean is diagnostic of osteoporosis [22].

**Fig. 1:**
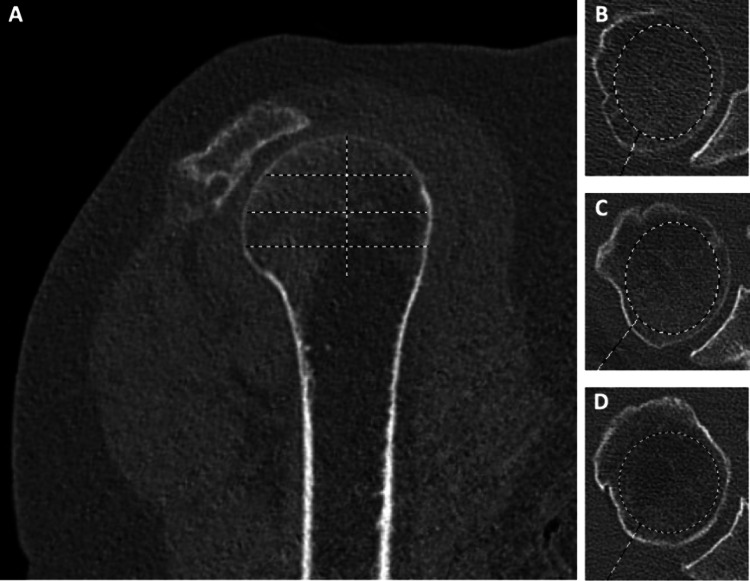
Example measurement of bone mineral density of the humeral head by CT method. A line is drawn on a sagittal cut from the superior humeral head to the surgical neck. This line is divided into quarters, with the dividing lines providing 3 axial slices. An ellipse is drawn on each identified axial cut to measure Houndsfield units (HU) excluding all cortical bone. The average of the 3 measurements is taken as the final value [24]

Numerous studies have highlighted the utility of computed tomography (CT) scans as an avenue to both correlate with DEXA results and to independently estimate BMD [23–25]. This can typically be done by a Hounsfield Units (HU) measurement in the proximal humerus or glenoid neck. Given the high proportion of patients undergoing shoulder surgery who have a pre-operative CT scan for surgical planning, additionally measuring HU to estimate BMD from this scan would be a simple way to identify patients with osteoporosis (Fig. 1).

**Fig. 2:**
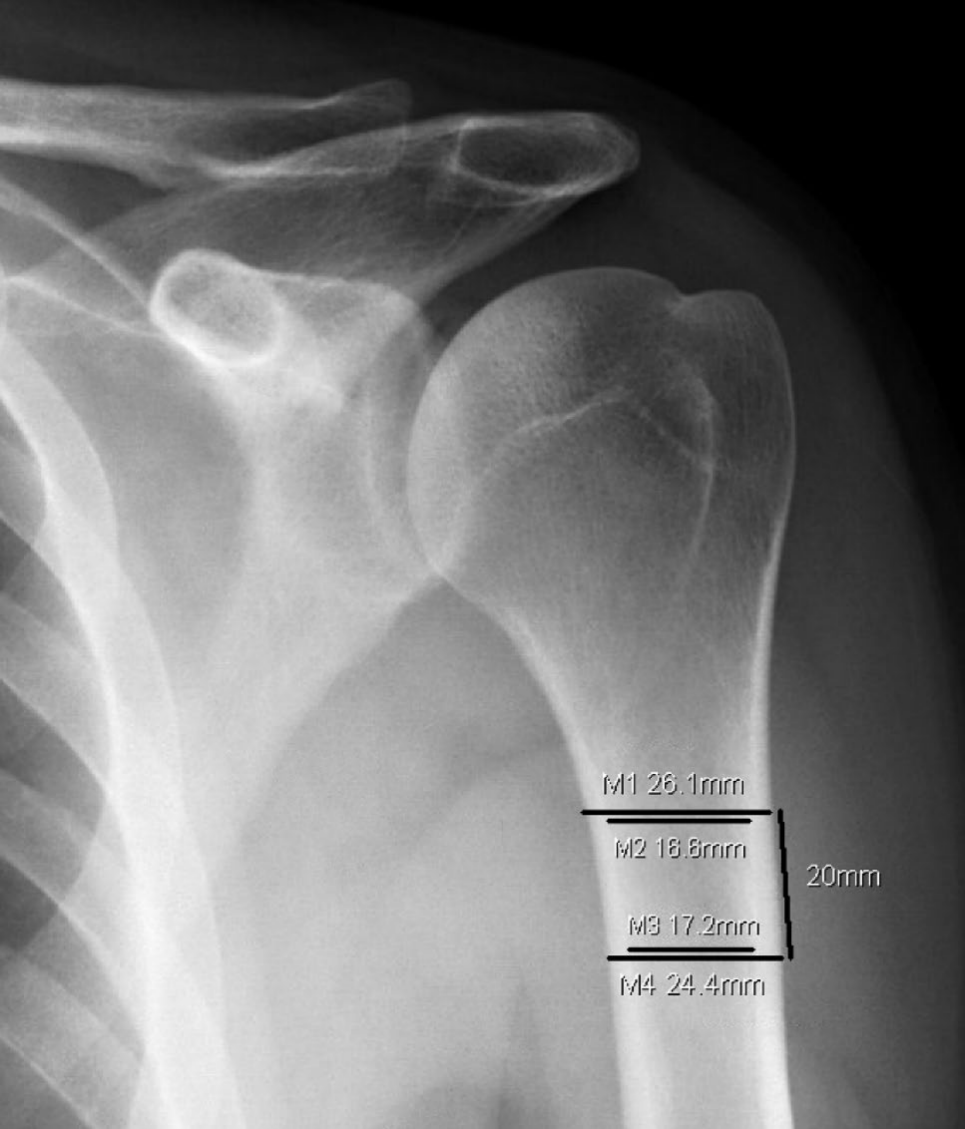
Example measurement of cortical bone thickness average. Cortical thickness is measured at two levels, 20 mm apart, with the proximal level being the first point at which the medial and lateral cortices become parallel. In this case the average of (M1-M2) and (M3-M4) is 8.35 [100]

**Fig. 3:**
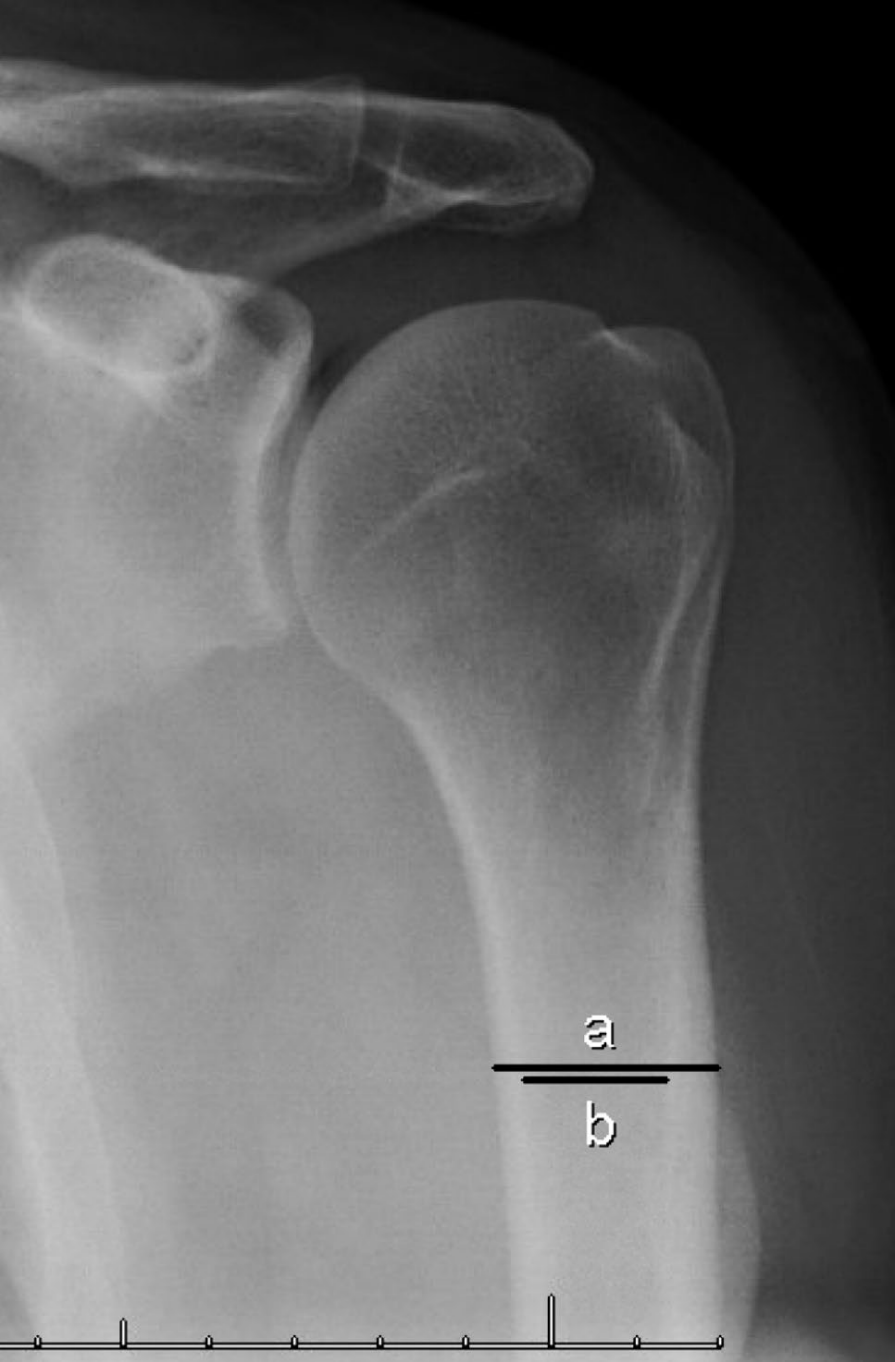
Example measurement of the deltoid tuberosity index. Cortical thickness for this measurement is taken at one site, immediately proximal to the deltoid tuberosity, and divided yielding a ratio (a/b) [28]

In addition to CT scans, plain films have been shown to have utility in assessing BMD for the proximal humerus. Cortical measurements from AP radiographs of the shoulder have shown an association with local BMD as well as with DEXA values for the femur, both by using the cortical bone thickness average (Fig. 2) or the deltoid tuberosity index (Fig. 3) [26–28]. With every shoulder surgery patient undergoing at least plain films preoperatively, this similarly presents a low cost, time-efficient way to screen for osteoporosis.

Despite the clear prevalence of osteoporosis and osteopenia in shoulder surgery patients and its detrimental effect on outcomes, there is minimal data on the advantages to pre-operative medical treatment in the form of bisphosphonates or anabolic agents. In one database study looking at patients with fragility fracture history undergoing shoulder arthroplasty, no difference was found between patients who took bisphosphonates pre-operatively and those who did not with regard to periprosthetic fracture and all-cause revision [12]. Moreover, one study has previously identified pre-operative bisphosphonate use as a risk factor for intraoperative fractures [29]. In terms of operatively treated proximal humerus fracture, Seo et al. identified no difference in union rates or clinical outcomes with early bisphosphonate initiation. [30] Lastly, one clinical study found that zoledronic acid improved rotator cuff healing with reduced retear rate [31] and decreased failure rates [32] in female patients with osteoporosis. However, a database study found no difference in revision rates of rotator cuff repairs amongst patients with osteoporosis who did or did not take bisphosphonates [33].

A more easily agreed upon pre-operative intervention would be vitamin D supplementation, due to the low risk and cost of its administration. Recommended dietary allowances are listed in Table 1 [34–36]. Vitamin D deficiency has been linked to post-operative complications in shoulder arthroplasty and rotator cuff repair.^84^ Similarly, it has been associated with a higher retear rate, revision rate, and early pain score after rotator cuff repair [37–39]. Due to this, vitamin D supplementation has been identified as a worthy adjunct in rotator cuff repair [40, 41].

**Table 1:** Recommended daily intake of calcium and vitamin D for adults. Recommendations include the total amount from all sources, both dietary and supplements. IU = International Units; mg = milligrams

Age	Women	Men
Calcium		
19–50	1000 mg	1000 mg
51–70	1200 mg	1000 mg
> 70	1200 mg	1200 mg
Vitamin D		
19–50	400–800 IU	400–800 IU
> 50	800–1000 IU	800–1000 IU

### Surgical Considerations

Despite appropriate patient screening, indications and pre-operative optimization, there will continue to be an increasing number of patients undergoing shoulder surgery with poor bone quality. Many of these are non-elective cases where optimization is not possible. In these cases, there are specific techniques and considerations that can be considered to optimize outcomes and minimize complications in patients with poor bone quality.

### Proximal Humerus Fracture Fixation

As with all osteoporotic fractures, proximal humerus fracture fixation is fraught with difficulties, including limited screw purchase, screw pullout, and failure of fixation. These complications happen despite the standard of care being contoured locking plates used as a fixed angle construct. Failure of fixation in these cases often presents as varus collapse, which can also be associated with screw cutout and penetration into the articular surface. This is a particularly morbid complication as it can lead to destruction of the glenoid articular surface [42]. Some of these complications can be solved by the use of locking screws, which is why a fixed angle construct is often considered the standard of care in osteoporotic bone. In addition, the surgeon should be aware of the locking mechanism of the screws they are using (threaded, variable angle, locking caps, differential material properties), to ensure that they are employed in the method intended and reduce the likelihood of malfunction or loss of fixation at the screw-plate interface.

**Fig. 4:**
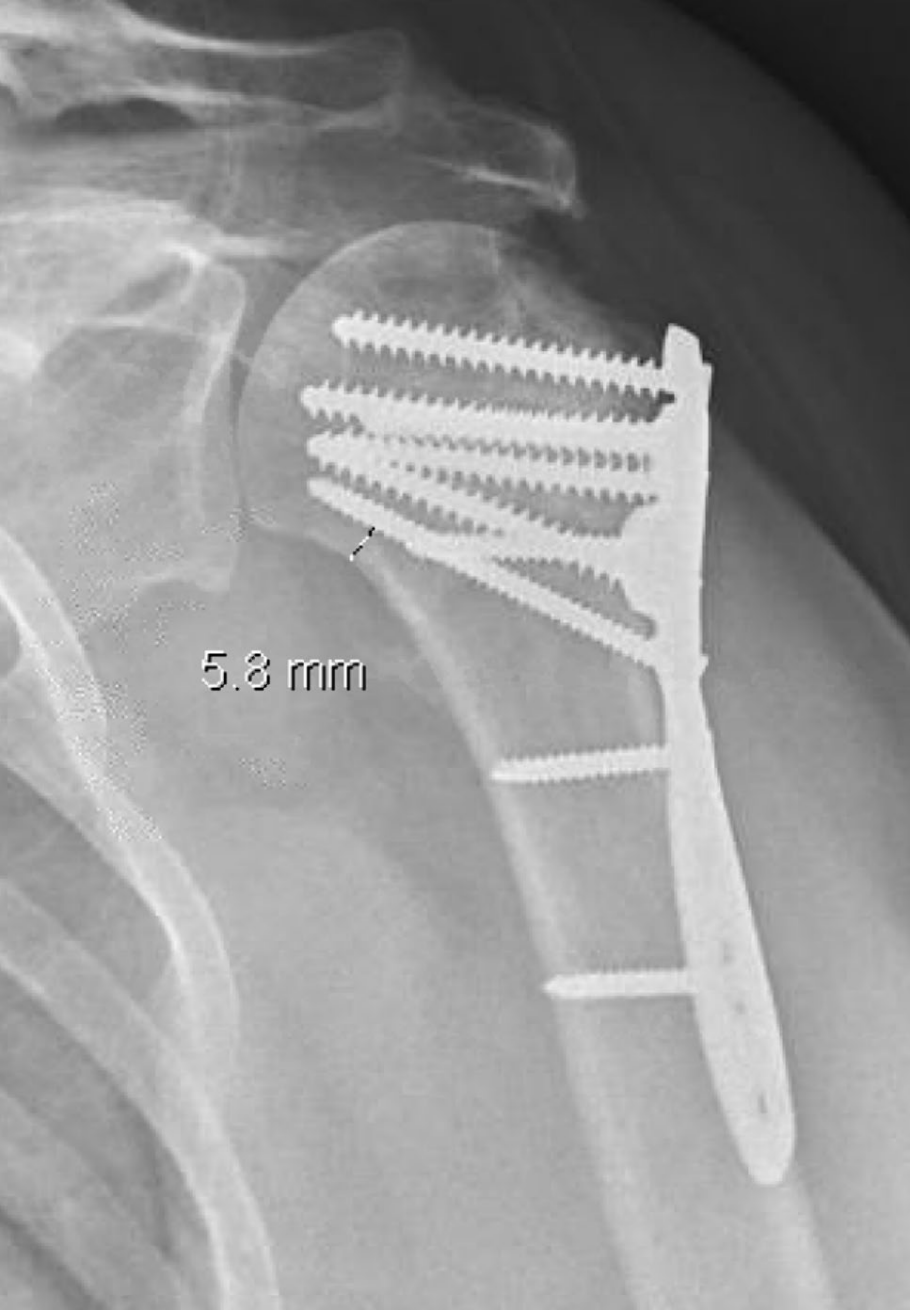
Example of a fixed angle locking plate used for fixation of a proximal humerus fracture, with measurement of the calcar screw distance demonstrated [44]

Screw positioning is another factor that has been studied in proximal humerus fracture fixation. Specifically, distal calcar screw placement compared to neutral placement in osteoporotic bone leads to increased torsional stiffness in external rotation, increased axial stiffness and decreased humeral head displacement during fatigue testing [43]. Optimal placement of the calcar screw was defined by Padegimas et al. as being within 12 mm of the calcar or with a ratio of the calcar distance and humeral head diameter of 25% (example measurement shown in Fig. 4). If these thresholds were not met, they found patients were at a higher risk for fixation failure and subsequent reoperation [44].

Although not routinely used, screw augmentation through cement or bone grafting has been shown to have favorable mechanics and outcomes. Polymethyl methacrylate (PMMA) cement has been shown to decrease the chance of fixation failure in low bone mineral density patients [45]. Similarly, there is support for calcium phosphate augmentation in osteopenic bone because it increases torsional and axial stiffness [46], improves compressive strength and structural support, [47] and decreases fracture displacement without screw penetration [48]. Cancellous autograft bone has also been used as an augment to plate fixation for multipart fractures and fractures with severe comminution and has been shown to minimize complications including avascular necrosis and nonunion [49, 50].

Suture augmentation is another technique with good literature support that is routinely used to augment fixation of the tuberosities and prevent tendon tearing or pullout in osteoporotic bone [4]. This can usually be accomplished by tying a suture configuration through holes in a plate, and can help avoid secondary tuberosity displacement [47]. It should be considered in cases with suboptimal reduction, multiple fracture fragments, comminution and when a plate only partially covers the greater tuberosity [47].

Additionally, a medial strut graft, most commonly performed with fibula allograft, can be used to augment internal fixation in osteoporotic bone as medial support to prevent varus collapse [51]. Studies have found that fibular strut grafts in osteoporotic bone result in lower intercyclic motion [52], lower fragment migration [52], less residual deformation [52], greater load to failure[53], increased stability [54], increased resistance to repetitive varus loading [55], and increased stiffness [54].

Another consideration is intramedullary nailing, which allows load sharing and reduces stress at the bone implant surface. This makes it an excellent option for treating osteoporotic proximal humerus fractures [56]. Although previous studies have shown some associated morbidity, including rotator cuff injury, shoulder pain, subacromial implant prominence, inadequate fixation and difficulty with reconstruction of nonunion; evolving contemporary techniques are reducing the incidence [57] of these complications [42]. More recent studies have shown no difference in complication rates between intramedullary nailing and locking plate fixation for proximal humerus fractures [57, 58]. The decision between intramedullary nailing versus plate fixation should be chosen based on the type of fracture, patient factors and surgeon experience, utilizing shared decision making between patient and surgeon [57, 58].

### Shoulder Arthroplasty for Fracture

Three and 4-part proximal humerus fractures in osteoporotic bone are considered to be at high risk for fixation failure or avascular necrosis. This reality combined with a growing body of literature supporting satisfactory outcomes for reverse shoulder arthroplasty performed for proximal humerus fractures has led to an increase in its utilization for this indication [59, 60].

Considerations for reverse total shoulder arthroplasty (RTSA) for repair of fracture in an osteoporotic patient includes stem positioning, tuberosity fixation, and cement utilization. Stem positioning and distalization is important for fixation and stability, because a shortened limb leads to instability and an increased incidence of dislocation, although balancing this with risk factors for scapular spine fractures must be considered [61]. In cases where a cortical read is not possible to assess height, the distance between the upper border of the pectoralis major tendon insertion and the top of the implant can be used for reconstruction of the humeral length for improved reduction and stability [62, 63].

Literature has shown that the successful healing of the greater tuberosity leads to improved external rotation after RTSA for fracture [64–66]. Cerclage fixation may cause less tuberosity interfragmentary rotation when compared to suture fixation, but has not been studied specifically in osteoporotic bones [67]. Autograft bone grafting of the tuberosities has been show to improve tuberosity healing, with greater disability of the arm, shoulder and hand (DASH) scores and American shoulder and elbow surgeons (ASES) scores [68–70].

Comparisons between cemented and uncemented stems for RTSA for fractures in osteoporotic bone have shown no difference in functional outcomes [71, 72]. One study found that cemented stems had higher rates of stem loosening than press fit stems [72]. Another study found that the incidence of radiographic signs of bone resorption was higher in uncemented RTSA, but was without clinical significance as both cemented and uncemented showed good functional results and low complications rates [71]. If using a cemented implant, keeping cement out of the metaphyseal region is expected to help with tuberosity healing [4].

Hemiarthroplasty (HA) is another option for management of proximal humeral fracture in patients with poor bone quality [73] or as an alternative arthroplasty in patients with glenoid bone deficiency or posterior wear [74]. Surgeons should consider that osteopenia is a risk factor for failure in HA [75]. Furthermore, healing from hemiarthroplasty is unreliable with an approximated 50% rate of tuberosity malposition [76]. Factors that contribute to this are improper prosthetic version and height and tuberosity mal-reduction that may stress the rotator cuff and hinder tuberosity healing [60].

### Elective Shoulder Arthroplasty

As previously described, low BMD has been shown to increase the incidence of both periprosthetic fractures [11, 12] and infection [12] after elective shoulder arthroplasty. Surgical considerations to mitigate fracture risk and improve implant fixation include careful retractor placement, cement utilization, and use of longer stems. [77],^24^ Stem selection is particularly important in osteoporotic bone, with some literature recommending increased stem diameter and length to ensure adequate fixation.^23,24^ Additionally, some studies have found that in osteoporotic bone, hand reaming should be used instead of power instruments to reduce the risk of removing too much cancellous bone and the chance of cortical perforation [78, 79]. Stemless implants for anatomic TSA were initially advised to be avoided in patients with osteoporosis; however, contemporary practice continues to evolve and expand the indications for stemless implants [80]. Furthermore, the generally accepted intraoperative thumb test to determine candidate suitability for a stemless implant has shown to be a poor indicator of low BMD.^37^

### Rotator Cuff Repair

In order to reduce incidence of re-tear or repair failure, surgeons should consider the bone density of the humerus, specifically the greater tuberosity, when planning rotator cuff surgery. Higher trabecular and cortical bone density were found in the proximal part of the tuberosities [16, 81, 82] and the humeral head has higher density then the neck [83]. As a result, these areas have been suggested as preferred anchor locations to prevent anchor loosening or pullout. Because osteoporotic patients have lower bone quality in the greater tuberosity, surgeons should limit or completely avoid aggressive decortications of the tuberosity region to preserve cortical bone [8].

In general, the more fixation points, the less stress concentrated over the anchor bone and suture tendon interface. This supports the use of a greater number of anchors to share the stress. [8] In the past, metal anchors have been a mainstay of rotator cuff repair; however, metal anchors were tested against newer bioabsorbable anchors in the osteoporotic patient, and bioabsorable anchors outperformed metal in pullout strength [84]. Additionally, bone screw anchors have increased pullout strength compared to hook type anchor in osteoporotic bone [81].

In cases of osteoporotic bone, both cement augmentation [85, 86] and magnesium phosphate augmentation [85] have been found to increase anchor pullout strength compared to conventional techniques. Previously, considering Burkhart’s Deadman angle [87] reduced the angle between suture anchor and rotator cuff tendon, resulting in higher pullout strength [84]. More recently, however, insertion angles of anchors were revisited. Strauss found 90 degree anchors had significantly improved anchor pullout strength than 45 degree, which was support by Eji et al. [88, 89].

### Utility of Osteoporosis Medications

The role for osteoporosis medications in fracture prevention is beyond the scope of this review, which will focus on specific indications for pharmacotherapy as they relate to shoulder surgery. Historically, the mainstay of medical treatment for osteoporosis has been bisphosphonates, which are considered to be anti-resorptive agents [90]. More recently, anabolic agents have become available with US Food and Drug Administration (FDA) approvals of abaloparatide (Tymlos) in 2022, romosozumab (Evenity) in 2019, and teriparatide (Forteo) in 2002. Animal studies have begun to show promising results for anabolic agents used to biologically augment tendon repairs, and increasing attention has been turned towards their utility in fracture healing.

As previously stated, pre-operative use of bisphosphonates have not been found to consistently enhance outcomes in shoulder arthroplasty, fracture fixation or rotator cuff repair, in the few clinical studies that have examined this. [12, 29–33]. One clinical study found that zoledronic acid improved rotator cuff healing with reduced retear rate [31] and decreased failure rates [32] in female patients with osteoporosis. However, a database study found no difference in revision rates of rotator cuff repairs amongst patients with osteoporosis who did or did not take bisphosphonates [33]. One case study has shown effectiveness of teriparatide used as an adjunct for successful non-operative treatment of post operative acromial stress fracture after RTSA, which may merit further investigation [91]. Anti-osteoporosis agents raloxifene, [92] risedronate, [93] zoledronic acid [94], teriparatide [95] and abaloparatide [96] have been studied in rotator cuff healing in rats and all showed promising results.

Finally, and perhaps most importantly in present day clinical practice, is the use of these medications to prevent secondary fractures. Within 5 years of a proximal humerus fracture, 20% of patients have been found to suffer a secondary fracture, with database data supporting less than 2% of patients with proximal humerus fractures receiving appropriate evaluation and treatment [97, 98]. Shoulder surgeons frequently treat patients with proximal humerus fragility fractures, and increasing resources make evaluation, treatment, or referral accessible in many (though not all) regions. Implementation of a fracture liaison service has been shown to increase osteoporosis screening in all cases of fragility fractures, as well as specifically patients who suffer proximal humerus fractures [99].

## Conclusions

As our population of increasing age with high functional demands continues to grow, we will increasingly perform shoulder surgery on patients with poor bone quality. Incorporating routine use of lifestyle and nutrition optimization, as well as opportunistic bone density screening into clinical practice, are simple steps to reduce surgical complications from low BMD. Intraoperative techniques should be modified when necessary to limit the risk of complications as well and improve implant longevity and outcomes. Further research is needed to identify the utility of pre-operative osteoporosis medications for patients with low BMD undergoing shoulder surgery; as well as clinical studies examining biologic augmentation with anabolic medications. Osteoporosis screening and referral should also have a low-barrier pathway for all shoulder surgeons to perform or direct their patients towards.

## Key References

- Chung SW, Oh JH, Gong HS, Kim JY, Kim SH. Factors Affecting Rotator Cuff Healing After Arthroscopic Repair: Osteoporosis as One of the Independent Risk Factors. Am J Sports Med. 2011;39:2099–107.
  - This article provides from context surrounding the concern for complications after rotator cuff repair in patients with osteoporosis.
- Casp AJ, Montgomery SR, Cancienne JM, Brockmeier SF, Werner BC. Osteoporosis and Implant-Related Complications After Anatomic and Reverse Total Shoulder Arthroplasty. J Am Acad Orthop Surg. 2020;28:121–7.
  - This article provides from context surrounding the concern for complications after shoulder arthroplasty in patients with osteoporosis.
- Cotter EJ, Klosterman EL, Winzenried AE, Greiner JJ, Grogan BF. Osteoporosis Screening Is Often Indicated but Overlooked Prior to Rotator Cuff Repair. Arthrosc Sports Med Rehabil. 2021;3:e659–65.
  - A review of epidemiology related to rotator cuff repair, highlighting the scope of this challenge.
- Bernatz JT, Brooks AE, Nguyen BP, Shin ED, Binkley NC, Anderson PA, et al. Prevalence and Treatment of Osteoporosis Prior to Elective Shoulder Arthroplasty. J Am Acad Orthop Surg Glob Res Rev. 2020;4:e20.00204.
  - A review of epidemiology related to shoulder arthroplasty, which highlights the scope of this challenge.
- Mather J, MacDermid JC, Faber KJ, Athwal GS. Proximal humerus cortical bone thickness correlates with bone mineral density and can clinically rule out osteoporosis. J Shoulder Elbow Surg. 2013;22:732–8.
  - This study presents one of several opportunistic methods to easily predict proximal humerus bone density using existing imaging.
- Mehta S, Chin M, Sanville J, Namdari S, Hast MW. Calcar screw position in proximal humerus fracture fixation: Don’t miss high! Injury. 2018;49:624–9.
  - An important technical consideration in proximal humerus fracture fixation is reviewed and quantified in this paper.
- Kramer M, Olach M, Zdravkovic V, Manser M, Jost B, Spross C. Cemented vs. uncemented reverse total shoulder arthroplasty for the primary treatment of proximal humerus fractures in the elderly—a retrospective case–control study. BMC Musculoskelet Disord. 2022;23:1043.
  - A common technical branch point in shoulder surgery patients with osteoporosis is discussed and examined in this study.
- Lei M, Zhu Z, Hu X, Wu D, Huang W, Zhang Y, et al. Postoperative Antiosteoporotic Treatment with Zoledronic Acid Improves Rotator Cuff Healing but Not Ameliorates Outcomes in Female Patients with Postmenopausal Osteoporosis: A Prospective, Single-Blinded Randomized Study. Arthrosc J Arthrosc Relat Surg Off Publ Arthrosc Assoc N Am Int Arthrosc Assoc. 2023;S0749-8063(23)00812–5.
  - One of several studies that reports outcomes in human patients after rotator cuff repair, when taking bisphosphonates post-operatively.
- Xu J, Ye Z, Chen C, Zhang X, Han K, Wu X, et al. Abaloparatide Improves Rotator Cuff Healing via Anabolic Effects on Bone Remodeling in a Chronic Rotator Cuff Tear Model of Rat With Osteoporosis: A Comparison With Denosumab. Am J Sports Med. 2022;50:1550–63.
  - This paper reports on the most recent investigation into anabolic anti-osteoporosis medications as a biologic augment to rotator cuff healing, in an animal model.

## Data Availability

No datasets were generated or analysed during the current study.

## References

[1] Office of the Surgeon General (US). Bone Health and Osteoporosis: A Report of the Surgeon General [Internet]. Rockville (MD): Office of the Surgeon General (US); 2004 [cited 2024 Jan 15]. Available from: http://www.ncbi.nlm.nih.gov/books/NBK45513/20945569

[2] Shen Y, Huang X, Wu J, Lin X, Zhou X, Zhu Z, et al. The Global Burden of Osteoporosis, Low Bone Mass, and Its Related Fracture in 204 Countries and Territories, 1990–2019. Front Endocrinol. 2022;13:882241. 10.3389/fendo.2022.882241 PMC9165055 35669691

[3] Pouresmaeili F, Kamalidehghan B, Kamarehei M, Goh YM. A comprehensive overview on osteoporosis and its risk factors. Ther Clin Risk Manag. 2018;14:2029–49. 30464484 10.2147/TCRM.S138000 PMC6225907

[4] Stone MA, Namdari S. Surgical Considerations in the Treatment of Osteoporotic Proximal Humerus Fractures. Orthop Clin North Am. 2019;50:223–31. 30850080 10.1016/j.ocl.2018.10.005

[5] Daher M, Fares MY, Boufadel P, Khanna A, Zalaquett Z, Abboud JA. Osteoporosis in the Setting of Shoulder Arthroplasty: A Narrative Review. Geriatr Orthop Surg Rehabil. 2023;14:21514593231182530. 37325699 10.1177/21514593231182527 PMC10265344

[6] Yakacki CM, Poukalova M, Guldberg RE, Lin A, Saing M, Gillogly S, et al. The Effect of the Trabecular Microstructure on the Pullout Strength of Suture Anchors. J Biomech. 2010;43:1953–9. 20399431 10.1016/j.jbiomech.2010.03.013 PMC2900467

[7] Oh LS, Wolf BR, Hall MP, Levy BA, Marx RG. Indications for rotator cuff repair: a systematic review. Clin Orthop. 2007;455:52–63. 17179786 10.1097/BLO.0b013e31802fc175

[8] Entezari V, Lazarus M. Surgical Considerations in Managing Osteoporosis, Osteopenia, and Vitamin D Deficiency During Arthroscopic Rotator Cuff Repair. Orthop Clin North Am. 2019;50:233–43. 30850081 10.1016/j.ocl.2018.10.006

[9] Chung SW, Oh JH, Gong HS, Kim JY, Kim SH. Factors Affecting Rotator Cuff Healing After Arthroscopic Repair: Osteoporosis as One of the Independent Risk Factors. Am J Sports Med. 2011;39:2099–107. 21813440 10.1177/0363546511415659

[10] Lee SH, Dargent-Molina P, Bréart G. Risk Factors for Fractures of the Proximal Humerus: Results From the EPIDOS Prospective Study. J Bone Miner Res. 2002;17:817–25. 12009012 10.1359/jbmr.2002.17.5.817

[11] Casp AJ, Montgomery SR, Cancienne JM, Brockmeier SF, Werner BC. Osteoporosis and Implant-Related Complications After Anatomic and Reverse Total Shoulder Arthroplasty. J Am Acad Orthop Surg. 2020;28:121–7. 31977612 10.5435/JAAOS-D-18-00537

[12] Testa EJ, Albright JA, Lemme NJ, Molla V, McCrae B, Daniels AH, et al. Increased Risk of Periprosthetic Fractures and Revision Arthroplasty in Patients Undergoing Shoulder Arthroplasty With a History of Prior Fragility Fractures: A Matched Cohort Analysis. J Am Acad Orthop Surg. 2023;31:e473–80. 36696566 10.5435/JAAOS-D-22-00752

[13] ASES Complications of RSA Research Group, Mahendraraj KA, Abboud J, Armstrong A, Austin L, Brolin T, et al. Predictors of acromial and scapular stress fracture after reverse shoulder arthroplasty: a study by the ASES Complications of RSA Multicenter Research Group. J Shoulder Elbow Surg. 2021;30:2296–305. 33677115 10.1016/j.jse.2021.02.008

[14] Mahendraraj KA, Abboud J, Armstrong A, Austin L, Brolin T, Entezari V, et al. Predictors of acromial and scapular stress fracture after reverse shoulder arthroplasty: a study by the ASES Complications of RSA Multicenter Research Group. J Shoulder Elbow Surg. 2021;30:2296–305. 33677115 10.1016/j.jse.2021.02.008

[15] Otto RJ, Virani NA, Levy JC, Nigro PT, Cuff DJ, Frankle MA. Scapular fractures after reverse shoulder arthroplasty: evaluation of risk factors and the reliability of a proposed classification. J Shoulder Elbow Surg. 2013;22:1514–21. 23659805 10.1016/j.jse.2013.02.007

[16] Tingart MJ, Bouxsein ML, Zurakowski D, Warner JP, Apreleva M. Three-dimensional distribution of bone density in the proximal humerus. Calcif Tissue Int. 2003;73:531–6. 14740644 10.1007/s00223-002-0013-9

[17] Kanis JA, Johnell O, Oden A, Johansson H, De Laet C, Eisman JA, et al. Smoking and fracture risk: a meta-analysis. Osteoporos Int J Establ Result Coop Eur Found Osteoporos Natl Osteoporos Found USA. 2005;16:155–62. 10.1007/s00198-004-1640-3 15175845

[18] Prieto-Alhambra D, Turkiewicz A, Reyes C, Timpka S, Rosengren B, Englund M. Smoking and Alcohol Intake but Not Muscle Strength in Young Men Increase Fracture Risk at Middle Age: A Cohort Study Linked to the Swedish National Patient Registry. J Bone Miner Res. 2020;35:498–504. 31714618 10.1002/jbmr.3917

[19] Diebo BG, Sheikh B, Freilich M, Shah NV, Redfern JAI, Tarabichi S, et al. Osteoporosis and Spine Surgery: A Critical Analysis Review. JBJS Rev. 2020;8:e0160. 33006455 10.2106/JBJS.RVW.19.00160

[20] Bernatz JT, Brooks AE, Nguyen BP, Shin ED, Binkley NC, Anderson PA, et al. Prevalence and Treatment of Osteoporosis Prior to Elective Shoulder Arthroplasty. J Am Acad Orthop Surg Glob Res Rev. 2020;4:e20.00204. 33986217 10.5435/JAAOSGlobal-D-20-00204 PMC7722598

[21] Cotter EJ, Klosterman EL, Winzenried AE, Greiner JJ, Grogan BF. Osteoporosis Screening Is Often Indicated but Overlooked Prior to Rotator Cuff Repair. Arthrosc Sports Med Rehabil. 2021;3:e659–65. 34195629 10.1016/j.asmr.2021.01.002 PMC8220567

[22] Kanis JA, McCloskey EV, Johansson H, Oden A, Melton LJ, Khaltaev N. A reference standard for the description of osteoporosis. Bone. 2008;42:467–75. 18180210 10.1016/j.bone.2007.11.001

[23] Nappo KE, Christensen DL, Wolfe JA, Tintle SM. Glenoid neck Hounsfield units on computed tomography can accurately identify patients with low bone mineral density. J Shoulder Elbow Surg. 2018;27:1268–74. 29397295 10.1016/j.jse.2017.11.008

[24] Pervaiz K, Cabezas A, Downes K, Santoni BG, Frankle MA. Osteoporosis and shoulder osteoarthritis: incidence, risk factors, and surgical implications. J Shoulder Elbow Surg. 2013;22:e1-8. 22938788 10.1016/j.jse.2012.05.029

[25] Werner BS, Hudek R, Burkhart KJ, Gohlke F. The influence of three-dimensional planning on decision-making in total shoulder arthroplasty. J Shoulder Elbow Surg. 2017;26:1477–83. 28162884 10.1016/j.jse.2017.01.006

[26] Handa A, Uchiyama Y, Shinpuku E, Watanabe M. Comparison of three plain radiography methods for evaluating proximal humerus bone strength in women. J Orthop Sci Off J Jpn Orthop Assoc. 2019;24:243–9. 10.1016/j.jos.2018.09.020 30361168

[27] Mather J, MacDermid JC, Faber KJ, Athwal GS. Proximal humerus cortical bone thickness correlates with bone mineral density and can clinically rule out osteoporosis. J Shoulder Elbow Surg. 2013;22:732–8. 23183030 10.1016/j.jse.2012.08.018

[28] Spross C, Kaestle N, Benninger E, Fornaro J, Erhardt J, Zdravkovic V, et al. Deltoid Tuberosity Index: A Simple Radiographic Tool to Assess Local Bone Quality in Proximal Humerus Fractures. Clin Orthop. 2015;473:3038–45. 25910780 10.1007/s11999-015-4322-x PMC4523505

[29] Mai DH, Oh C, Doany ME, Rokito AS, Kwon YW, Zuckerman JD, et al. Preoperative bisphosphonate treatment may adversely affect the outcome after shoulder arthroplasty. Bone Jt J. 2019;101-B:147–53. 10.1302/0301-620X.101B2.BJJ-2018-0906.R1 30700113

[30] Seo J-B, Yoo J-S, Ryu J-W, Yu K-W. Influence of Early Bisphosphonate Administration for Fracture Healing in Patients with Osteoporotic Proximal Humerus Fractures. Clin Orthop Surg. 2016;8:437–43. 27904727 10.4055/cios.2016.8.4.437 PMC5114257

[31] Lei M, Zhu Z, Hu X, Wu D, Huang W, Zhang Y, et al. Postoperative Antiosteoporotic Treatment with Zoledronic Acid Improves Rotator Cuff Healing but Not Ameliorates Outcomes in Female Patients with Postmenopausal Osteoporosis: A Prospective, Single-Blinded Randomized Study. Arthrosc J Arthrosc Relat Surg Off Publ Arthrosc Assoc N Am Int Arthrosc Assoc. 2023;S0749–8063(23):00812–5. 10.1016/j.arthro.2023.09.033 37832742

[32] Lee J-H, Yoon J-Y, Lee Y-B. The Use of Intravenous Zoledronate May Reduce Retear Rate after Rotator Cuff Repair in Older Female Patients with Osteoporosis: A First In-Human Prospective Study. J Clin Med. 2022;11:836. 35160287 10.3390/jcm11030836 PMC8836943

[33] Cancienne JM, Brockmeier SF, Kew ME, Deasey MJ, Werner BC. The Association of Osteoporosis and Bisphosphonate Use With Revision Shoulder Surgery After Rotator Cuff Repair. Arthrosc J Arthrosc Relat Surg Off Publ Arthrosc Assoc N Am Int Arthrosc Assoc. 2019;35:2314–20. 10.1016/j.arthro.2019.03.036 31231005

[34] Calcium/Vitamin D Requirements, Recommended Foods & Supplements [Internet]. 2015 [cited 2024 Jul 1]. Available from: https://www.bonehealthandosteoporosis.org/patients/treatment/calciumvitamin-d/

[35] Office of Dietary Supplements - Calcium [Internet]. [cited 2024 Jul 1]. Available from: https://ods.od.nih.gov/factsheets/Calcium-HealthProfessional/

[36] Office of Dietary Supplements - Vitamin D [Internet]. [cited 2024 Jul 1]. Available from: https://ods.od.nih.gov/factsheets/VitaminD-HealthProfessional/

[37] Cancienne JM, Brockmeier SF, Kew ME, Werner BC. Perioperative Serum 25-Hydroxyvitamin D Levels Affect Revision Surgery Rates After Arthroscopic Rotator Cuff Repair. Arthrosc J Arthrosc Relat Surg Off Publ Arthrosc Assoc N Am Int Arthrosc Assoc. 2019;35:763–9. 10.1016/j.arthro.2018.09.032 30704888

[38] Chen J, Lou J, Wang W, Xu G. Association of Preoperative Vitamin D Deficiency With Retear Rate and Early Pain After Arthroscopic Rotator Cuff Repair: A Retrospective Cohort Study. Orthop J Sports Med. 2022;10:23259671221130316. 36276423 10.1177/23259671221130315 PMC9580096

[39] Harada GK, Arshi A, Fretes N, Formanek B, Gamradt S, McAllister DR, et al. Preoperative Vitamin D Deficiency Is Associated With Higher Postoperative Complications in Arthroscopic Rotator Cuff Repair. J Am Acad Orthop Surg Glob Res Rev. 2019;3:e075. 31579883 10.5435/JAAOSGlobal-D-19-00075 PMC6743985

[40] Patel D, Roy G, Endres N, Ziino C. Preoperative vitamin D supplementation is a cost-effective intervention in arthroscopic rotator cuff repair. J Shoulder Elbow Surg. 2023;32:2473–82. 37308074 10.1016/j.jse.2023.05.007

[41] Smith JM, Cancienne JM, Brockmeier SF, Werner BC. Vitamin D deficiency and total shoulder arthroplasty complications. Shoulder Elb. 2021;13:99–105. 10.1177/1758573220906520 PMC7905506 33717223

[42] Ring D. Current concepts in plate and screw fixation of osteoporotic proximal humerus fractures. Injury. 2007;38:59–68. 10.1016/j.injury.2007.08.013 17723794

[43] Mehta S, Chin M, Sanville J, Namdari S, Hast MW. Calcar screw position in proximal humerus fracture fixation: Don’t miss high! Injury. 2018;49:624–9. 29452734 10.1016/j.injury.2018.02.007 PMC7413303

[44] Padegimas EM, Zmistowski B, Lawrence C, Palmquist A, Nicholson TA, Namdari S. Defining optimal calcar screw positioning in proximal humerus fracture fixation. J Shoulder Elbow Surg. 2017;26:1931–7. 28688933 10.1016/j.jse.2017.05.003

[45] Unger S, Erhart S, Kralinger F, Blauth M, Schmoelz W. The effect of in situ augmentation on implant anchorage in proximal humeral head fractures. Injury. 2012;43:1759–63. 22824159 10.1016/j.injury.2012.07.003

[46] Hast MW, Chin M, Schmidt EC, Sanville J, Van Osten GK, Mehta S. Mechanical Effects of Bone Substitute and Far-Cortical Locking Techniques in 2-Part Proximal Humerus Fracture Reconstruction: A Cadaveric Study. J Orthop Trauma. 2020;34:199. 32197036 10.1097/BOT.0000000000001668

[47] Hente R, Kampshoff J, Kinner B, Füchtmeier B. Nerlich M [Treatment of dislocated 3- and 4-part fractures of the proximal humerus with an angle-stabilizing fixation plate]. Unfallchirurg. 2004;107:769–82. 15292960 10.1007/s00113-004-0818-7

[48] Egol KA, Sugi MT, Ong CC, Montero N, Davidovitch R, Zuckerman JD. Fracture site augmentation with calcium phosphate cement reduces screw penetration after open reduction–internal fixation of proximal humeral fractures. J Shoulder Elbow Surg. 2012;21:741–8. 22192764 10.1016/j.jse.2011.09.017

[49] Kim SH, Lee YH, Chung SW, Shin SH, Jang WY, Gong HS, et al. Outcomes for four-part proximal humerus fractures treated with a locking compression plate and an autologous iliac bone impaction graft. Injury. 2012;43:1724–31. 22819250 10.1016/j.injury.2012.06.029

[50] Ricchetti ET, Warrender WJ, Abboud JA. Use of locking plates in the treatment of proximal humerus fractures. J Shoulder Elbow Surg. 2010;19:66–75. 20188270 10.1016/j.jse.2010.01.001

[51] Gardner MJ, Weil Y, Barker JU, Kelly BT, Helfet DL, Lorich DG. The importance of medial support in locked plating of proximal humerus fractures. J Orthop Trauma. 2007;21:185–91. 17473755 10.1097/BOT.0b013e3180333094

[52] Osterhoff G, Baumgartner D, Favre P, Wanner GA, Gerber H, Simmen H-P, et al. Medial support by fibula bone graft in angular stable plate fixation of proximal humeral fractures: an in vitro study with synthetic bone. J Shoulder Elbow Surg. 2011;20:740–6. 21330155 10.1016/j.jse.2010.10.040

[53] Bae J-H, Oh J-K, Chon C-S, Oh C-W, Hwang J-H, Yoon Y-C. The biomechanical performance of locking plate fixation with intramedullary fibular strut graft augmentation in the treatment of unstable fractures of the proximal humerus. J Bone Joint Surg Br. 2011;93-B:937–41. 10.1302/0301-620X.93B7.26125 21705567

[54] Rusimov L, Zderic I, Ciric D, Barcik JP, Enchev D, Rashkov M, et al. Does Supplemental Intramedullary Grafting Increase Stability of Plated Proximal Humerus Fractures? J Orthop Trauma. 2019;33:196. 30570617 10.1097/BOT.0000000000001376

[55] Chow RM, Begum F, Beaupre LA, Carey JP, Adeeb S, Bouliane MJ. Proximal humeral fracture fixation: locking plate construct ± intramedullary fibular allograft. J Shoulder Elbow Surg. 2012;21:894–901. 21782474 10.1016/j.jse.2011.04.015

[56] Cornell CN. Internal Fracture Fixation in Patients With Osteoporosis. JAAOS - J Am Acad Orthop Surg. 2003;11:109. 12670137 10.5435/00124635-200303000-00005

[57] Boadi PJ, Da Silva A, Mizels J, Joyce CD, Anakwenze OA, Klifto CS, et al. Intramedullary versus locking plate fixation for proximal humerus fractures: indications and technical considerations. JSES Rev Rep Tech [Internet]. 2024 [cited 2024 May 30]; Available from: https://www.sciencedirect.com/science/article/pii/S2666639124000154 10.1016/j.xrrt.2024.01.001 PMC11329022 39157214

[58] Chen B-K, Tai T-H, Lin S-H, Chen K-H, Huang Y-M, Chen C-Y. Intramedullary Nail vs. Plate Fixation for Pathological Humeral Shaft Fracture: An Updated Narrative Review and Meta-Analysis of Surgery-Related Factors. J Clin Med. 2024;13:755. 38337449 10.3390/jcm13030755 PMC10856436

[59] Longo UG, Petrillo S, Berton A, Denaro V. Reverse total shoulder arthroplasty for the management of fractures of the proximal humerus: a systematic review. Musculoskelet Surg. 2016;100:83–91. 27316439 10.1007/s12306-016-0409-0

[60] Acevedo DC, VanBeek C, Lazarus MD, Williams GR, Abboud JA. Reverse shoulder arthroplasty for proximal humeral fractures: update on indications, technique, and results. J Shoulder Elbow Surg. 2014;23:279–89. 24418780 10.1016/j.jse.2013.10.003

[61] Lädermann A, Williams MD, Melis B, Hoffmeyer P, Walch G. Objective evaluation of lengthening in reverse shoulder arthroplasty. J Shoulder Elbow Surg. 2009;18:588–95. 19481476 10.1016/j.jse.2009.03.012

[62] Cagle PJ, Reizner W, Parsons BO. A technique for humeral prosthesis placement in reverse total shoulder arthroplasty for fracture. Shoulder Elb. 2019;11:459–64. 10.1177/1758573218793904 PMC7094067 32269606

[63] Murachovsky J, Ikemoto RY, Nascimento LGP, Fujiki EN, Milani C, Warner JJP. Pectoralis major tendon reference (PMT): A new method for accurate restoration of humeral length with hemiarthroplasty for fracture. J Shoulder Elbow Surg. 2006;15:675–8. 17055748 10.1016/j.jse.2005.12.011

[64] Anakwenze OA, Zoller S, Ahmad CS, Levine WN. Reverse shoulder arthroplasty for acute proximal humerus fractures: a systematic review. J Shoulder Elbow Surg. 2014;23:e73-80. 24406120 10.1016/j.jse.2013.09.012

[65] Chun Y-M, Kim D-S, Lee D-H, Shin S-J. Reverse shoulder arthroplasty for four-part proximal humerus fracture in elderly patients: can a healed tuberosity improve the functional outcomes? J Shoulder Elbow Surg. 2017;26:1216–21. 28162882 10.1016/j.jse.2016.11.034

[66] Gallinet D, Adam A, Gasse N, Rochet S, Obert L. Improvement in shoulder rotation in complex shoulder fractures treated by reverse shoulder arthroplasty. J Shoulder Elbow Surg. 2013;22:38–44. 22705317 10.1016/j.jse.2012.03.011

[67] Knierzinger D, Heinrichs CH, Hengg C, Konschake M, Kralinger F, Schmoelz W. Biomechanical evaluation of cable and suture cerclages for tuberosity reattachment in a 4-part proximal humeral fracture model treated with reverse shoulder arthroplasty. J Shoulder Elbow Surg. 2018;27:1816–23. 29779978 10.1016/j.jse.2018.04.003

[68] Uzer G, Yildiz F, Batar S, Binlaksar R, Elmadag M, Kus G, et al. Does grafting of the tuberosities improve the functional outcomes of proximal humeral fractures treated with reverse shoulder arthroplasty? J Shoulder Elbow Surg. 2017;26:36–41. 27496351 10.1016/j.jse.2016.05.005

[69] Formaini NT, Everding NG, Levy JC, Rosas S. Tuberosity healing after reverse shoulder arthroplasty for acute proximal humerus fractures: the “black and tan” technique. J Shoulder Elbow Surg. 2015;24:e299-306. 26141197 10.1016/j.jse.2015.04.014

[70] Levy JC, Badman B. Reverse shoulder prosthesis for acute four-part fracture: Tuberosity fixation using a horseshoe graft. J Orthop Trauma. 2011;25:318–24. 21464740 10.1097/BOT.0b013e3181f22088

[71] Kramer M, Olach M, Zdravkovic V, Manser M, Jost B, Spross C. Cemented vs. uncemented reverse total shoulder arthroplasty for the primary treatment of proximal humerus fractures in the elderly—a retrospective case–control study. BMC Musculoskelet Disord. 2022;23:1043. 36457072 10.1186/s12891-022-05994-3 PMC9714093

[72] Lopiz Y, García-Fernandez C, Vallejo-Carrasco M, Garriguez-Pérez D, Achaerandio L, Tesoro-Gonzalo C, et al. Reverse shoulder arthroplasty for proximal humeral fracture in the elderly. Cemented or uncemented stem? Int Orthop. 2022;46:635–44. 35034145 10.1007/s00264-021-05284-y

[73] Grassi FA, Alberio R, Ratti C, Surace MF, Piazza P, Messinese P, et al. Shoulder arthroplasty for proximal humerus fractures in the elderly: The path from Neer to Grammont. Orthop Rev. 2020;12:8659. 10.4081/or.2020.8659 PMC7459385 32913595

[74] Hsu JE, Ricchetti ET, Huffman GR, Iannotti JP, Glaser DL. Addressing glenoid bone deficiency and asymmetric posterior erosion in shoulder arthroplasty. J Shoulder Elbow Surg. 2013;22:1298–308. 23796384 10.1016/j.jse.2013.04.014

[75] Borade AU, Familiari F, Choi K, Joseph J, McFarland EG. Comparison of Reverse Total Shoulder Arthroplasty vs Hemiarthroplasty for Acute Fractures of the Proximal Humerus: Systematic Review. J Postgrad Med Educ Res. 2017;51:182–7.

[76] Boileau P, Krishnan SG, Tinsi L, Walch G, Coste JS, Molé D. Tuberosity malposition and migration: Reasons for poor outcomes after hemiarthroplasty for displaced fractures of the proximal humerus. J Shoulder Elbow Surg. 2002;11:401–12. 12378157 10.1067/mse.2002.124527

[77] Cronin KJ, Vaughan A, Tzeuton S, Abboud JA. Prospective assessment of osteoporosis in total shoulder arthroplasty. Semin Arthroplasty JSES. 2023;33:15–21.

[78] Wirth MA, Rockwood CA Jr. Current Concepts Review - Complications of Total Shoulder-Replacement Arthroplasty*. JBJS. 1996;78:603. 10.2106/00004623-199604000-00018 8609143

[79] Friedman RJ. Humeral Technique in Total Shoulder Arthroplasty. Orthop Clin North Am. 1998;29:393–402. 9706286 10.1016/s0030-5898(05)70015-8

[80] Hawi N, Tauber M, Messina MJ, Habermeyer P, Martetschläger F. Anatomic stemless shoulder arthroplasty and related outcomes: a systematic review. BMC Musculoskelet Disord. 2016;17:376. 27577859 10.1186/s12891-016-1235-0 PMC5006279

[81] Tingart MJ, Apreleva M, Lehtinen J, Zurakowski D, Warner JJP. Anchor design and bone mineral density affect the pull-out strength of suture anchors in rotator cuff repair: which anchors are best to use in patients with low bone quality? Am J Sports Med. 2004;32:1466–73. 15310572 10.1177/0363546503262644

[82] Kirchhoff C, Braunstein V, Milz S, Sprecher CM, Fischer F, Tami A, et al. Assessment of bone quality within the tuberosities of the osteoporotic humeral head: relevance for anchor positioning in rotator cuff repair. Am J Sports Med. 2010;38:564–9. 20118499 10.1177/0363546509354989

[83] Saitoh S, Nakatsuchi Y, Latta L, Milne E. Distribution of bone mineral density and bone strength of the proximal humerus. J Shoulder Elbow Surg. 1994;3:234–42. 22959752 10.1016/S1058-2746(09)80041-4

[84] Pietschmann MF, Fröhlich V, Ficklscherer A, Gülecyüz MF, Wegener B, Jansson V, et al. Suture anchor fixation strength in osteopenic versus non-osteopenic bone for rotator cuff repair. Arch Orthop Trauma Surg. 2009;129:373–9. 18607610 10.1007/s00402-008-0689-4

[85] Braunstein V, Ockert B, Windolf M, Sprecher CM, Mutschler W, Imhoff A, et al. Increasing pullout strength of suture anchors in osteoporotic bone using augmentation–a cadaver study. Clin Biomech Bristol Avon. 2015;30:243–7. 25686676 10.1016/j.clinbiomech.2015.02.002

[86] AlThani S, Meshram P. Cement Augmentation of Suture Anchor During Arthroscopic Rotator Cuff Repair in Case of Proximal Humeral Bone Deficiency Due to Osteoporosis. Arthrosc Tech. 2023;12:e897-902. 37424634 10.1016/j.eats.2023.02.025 PMC10323824

[87] Burkhart SS. The deadman theory of suture anchors: observations along a south Texas fence line. Arthrosc J Arthrosc Relat Surg Off Publ Arthrosc Assoc N Am Int Arthrosc Assoc. 1995;11:119–23. 10.1016/0749-8063(95)90100-0 7727005

[88] Itoi E, Nagamoto H, Sano H, Yamamoto N, Kawakami J. Deadman theory revisited. Biomed Mater Eng. 2016;27:171–81. 27567773 10.3233/BME-161586

[89] Strauss E, Frank D, Kubiak E, Kummer F, Rokito A. The Effect of the Angle of Suture Anchor Insertion on Fixation Failure at the Tendon-Suture Interface After Rotator Cuff Repair: Deadman’s Angle Revisited. Arthrosc J Arthrosc Relat Surg. 2009;25:597–602. 10.1016/j.arthro.2008.12.021 19501288

[90] Reid IR, Billington EO. Drug therapy for osteoporosis in older adults. The Lancet. 2022;399:1080–92. 10.1016/S0140-6736(21)02646-5 35279261

[91] Lipof JS, Southgate RD, Tyler WK, Bukata SV, Voloshin I. Treatment of an Acromial Stress Fracture After Reverse Total Shoulder Arthroplasty With Teriparatide: A Case Report. JBJS Case Connect. 2020;10:e0221. 32649098 10.2106/JBJS.CC.19.00221

[92] Kim DM, Shim IK, Shin MJ, Choi JH, Lee YN, Jeon I-H, et al. A Combination Treatment of Raloxifene and Vitamin D Enhances Bone-to-Tendon Healing of the Rotator Cuff in a Rat Model. Am J Sports Med. 2020;48:2161–9. 32574070 10.1177/0363546520927015

[93] Xu J, Su W, Chen J, Ye Z, Wu C, Jiang J, et al. The Effect of Antiosteoporosis Therapy With Risedronate on Rotator Cuff Healing in an Osteoporotic Rat Model. Am J Sports Med. 2021;49:2074–84. 33998839 10.1177/03635465211011748

[94] Schanda JE, Keibl C, Heimel P, Monforte X, Tangl S, Feichtinger X, et al. Zoledronic Acid Substantially Improves Bone Microarchitecture and Biomechanical Properties After Rotator Cuff Repair in a Rodent Chronic Defect Model. Am J Sports Med. 2020;48:2151–60. 32543880 10.1177/0363546520926471

[95] Chen X, Giambini H, Ben-Abraham E, An K-N, Nassr A, Zhao C. Effect of Bone Mineral Density on Rotator Cuff Tear: An Osteoporotic Rabbit Model. PLoS ONE. 2015;10:e0139384. 26466092 10.1371/journal.pone.0139384 PMC4605490

[96] Xu J, Ye Z, Chen C, Zhang X, Han K, Wu X, et al. Abaloparatide Improves Rotator Cuff Healing via Anabolic Effects on Bone Remodeling in a Chronic Rotator Cuff Tear Model of Rat With Osteoporosis: A Comparison With Denosumab. Am J Sports Med. 2022;50:1550–63. 35404150 10.1177/03635465221079651

[97] Katthagen JC, Koeppe J, Stolberg-Stolberg J, Rischen R, Freistuehler M, Faldum A, et al. Effects of anti-osteoporosis therapy on the risk of secondary fractures and surgical complications following surgical fixation of proximal humerus fracture in older people. Age Ageing. 2023;52:afad097. 37368870 10.1093/ageing/afad097

[98] Kim TI, Choi JH, Kim SH, Oh JH. The Adequacy of Diagnosis and Treatment for Osteoporosis in Patients with Proximal Humeral Fractures. Clin Orthop Surg. 2016;8:274–9. 27583110 10.4055/cios.2016.8.3.274 PMC4987311

[99] Seyok T, Collins JE, Erikson SJ, Charles JF, Earp BE. Impact of an Outpatient Fracture Liaison Service on Osteoporosis Evaluation Among Patients With Upper Extremity Fragility Fracture. Hand N Y N. 2024;19:256–62. 10.1177/15589447221120851 PMC10953516 36113071

[100] Tingart MJ, Apreleva M, von Stechow D, Zurakowski D, Warner JJ. The cortical thickness of the proximal humeral diaphysis predicts bone mineral density of the proximal humerus. J Bone Joint Surg Br. 2003;85:611–7. 12793573 10.1302/0301-620x.85b4.12843

